# Molecular Mechanisms of Ursodeoxycholic Acid Toxicity & Side Effects: Ursodeoxycholic Acid Freezes Regeneration & Induces Hibernation Mode

**DOI:** 10.3390/ijms13078882

**Published:** 2012-07-17

**Authors:** Magd A. Kotb

**Affiliations:** Department of Pediatrics, Faculty of Medicine, Cairo University, Al-Saray Street, El Manial, Cairo 11956, Egypt; E-Mail: magdkotb@hotmail.com; Tel.: +202-2366-7260

**Keywords:** ursodeoxycholic acid, primary biliary cirrhosis, neonatal cholestasis, vanishing bile duct syndrome, toxicity, side effects, primary sclerosing cholangitis, PSC, extrahepatic biliary atresia, neonatal hepatitis

## Abstract

Ursodeoxycholic acid (UDCA) is a steroid bile acid approved for primary biliary cirrhosis (PBC). UDCA is reported to have “hepato-protective properties”. Yet, UDCA has “unanticipated” toxicity, pronounced by more than double number of deaths, and eligibility for liver transplantation compared to the control group in 28 mg/kg/day in primary sclerosing cholangitis, necessitating trial halt in North America. UDCA is associated with increase in hepatocellular carcinoma in PBC especially when it fails to achieve biochemical response (10 and 15 years incidence of 9% and 20% respectively). “Unanticipated” UDCA toxicity includes hepatitis, pruritus, cholangitis, ascites, vanishing bile duct syndrome, liver cell failure, death, severe watery diarrhea, pneumonia, dysuria, immune-suppression, mutagenic effects and withdrawal syndrome upon sudden halt. UDCA inhibits DNA repair, co-enzyme A, cyclic AMP, p53, phagocytosis, and inhibits induction of nitric oxide synthatase. It is genotoxic, exerts aneugenic activity, and arrests apoptosis even after cellular phosphatidylserine externalization. UDCA toxicity is related to its interference with drug detoxification, being hydrophilic and anti-apoptotic, has a long half-life, has transcriptional mutational abilities, down-regulates cellular functions, has a very narrow difference between the recommended (13 mg/kg/day) and toxic dose (28 mg/kg/day), and it typically transforms into lithocholic acid that induces DNA strand breakage, it is uniquely co-mutagenic, and promotes cell transformation. UDCA beyond PBC is unjustified.

## 1. Introduction

Ursodeoxycholic acid (UDCA) is a physiologic hydrophilic dihydroxy bile acid, which was first characterized in the bile of the Chinese black bear [1], and is present in man in a concentration of about 3% of the bile acid pool [2].

UDCA was Food and Drug Administration (FDA) approved for cholesterol gall stone dissolution, and primary biliary cirrhosis (PBC). UDCA is reported to increase bile flow, change the hydrophobicity index of the bile acid pool and has immune-suppressive effects [3–5].

UDCA does not affect long-term survival, or transplant free survival and does not slow progression of the PBC, but achieves a 25% drop in serum bilirubin, a 35% drop in serum alanine aminotransferase, a 33% drop in aspartate aminotrasferase, 40% drop in alkaline phosphatase and a 50% drop in gamma glutamyl transpeptidase, that is not associated with control of pruritus, fatigue or weakness. Evidence is lacking to support effectiveness of UDCA in PBC, beyond the control of serum bilirubin, and hepatic transaminases [6–9].

Beyond PBC, UDCA was studied as an investigational medication in a wide range of hepatic and extra-hepatic diseases. UDCA is not licensed for use in children. Its effectiveness and safety in the pediatric age group was never established [10]. Among a pediatric cohort with cholestasis studied in retrospect, of 734 infants with neonatal hepatitis, paucity of intrahepatic biliary radicals, and extrahepatic biliary atresia, 144/401 (35.9%) infants and children on UDCA achieved complete resolution of cholestasis and 236 (58.8%) failed to resolve cholestasis. Among the 333 who did not receive UDCA 218 (65.5%) achieved complete resolution of cholestasis and 30.3% did not resolve their cholestasis. UDCA use in neonatal and infancy cholestasis was associated with more than double fold the risk of failure of resolution of cholestasis, and life threatening complications, liver cell failure and death. Those who received UDCA were age, sex and etiology matched [11,12]. UDCA use in neonatal and infancy cholestasis was reported ineffective and unsafe warranting the halt of off-label use in unapproved indications of UDCA and the halt of a trial of UDCA in infants and children in Cairo University Children Hospitals by The Higher Committee For Medications-Cairo University Hospitals, in November 2010. UDCA in 15mg/kg/day is ineffective in primary sclerosing cholangitis, and upon doubling the dose (28–30 mg/kg/day); it was found ineffective, unsafe and detrimental necessitating halt of trial in North America. More than double number of patients developed varices, died, or became eligible for liver transplantation in the group receiving UDCA compared to placebo group (*p* = 0.01) [13]. In patients with primary sclerosing cholangitis treated with UDCA, dominant stenoses are associated with reduced survival free of liver transplantation and the role of inflammatory bowel disease in such patients is unclear [14].

Moreover, Rudolph and coworkers reported that after the start of UDCA in primary sclerosing cholangitis, the annual incidence of colorectal carcinoma increased up to 6 years and subsequently decreased. In primary sclerosing cholangitis with inflammatory bowel disease treated with UDCA, most colonic carcinomas develop in the first years after the start of treatment [15].

This tumorgenic property of UDCA is not unique to primary sclerosing cholangitis. In patients with PBC on UDCA, incidence of hepatocellular carcinoma (HCC) increases. The risk for HCC was highest in the group of non-responders to UDCA: the 10 years incidence of HCC was 9% and the 15 years incidence was 20%. It is essential to highlight that UDCA non-responders were defined as those who did not normalize serum bilirubin and albumin concentrations after 1 year of UDCA therapy, or those who did not retain their normal bilirubin level after 1 year of UDCA therapy [16]. Biochemical non-responders are almost 40% of treated PBC patients [17,18].

UDCA toxicity profile includes fever, hepatitis, cholangitis, vanishing bile duct syndrome, liver cell failure, death, severe watery diarrhea, pneumonia, interstitial lung disease, convulsions and mutagenic effects [12,13,19,20]. The likelihood of developing adverse events was not predicted by UDCA ability to control liver enzymes, or presence of cirrhosis on entry biopsy [12,13].

This review aims to define UDCA properties as a bile acid, its potential role, its molecular toxicities and lessons learned from “unanticipated” ineffectiveness and deaths of the recently halted trial [13].

## 2. UDCA Structure

**Figure 1:**
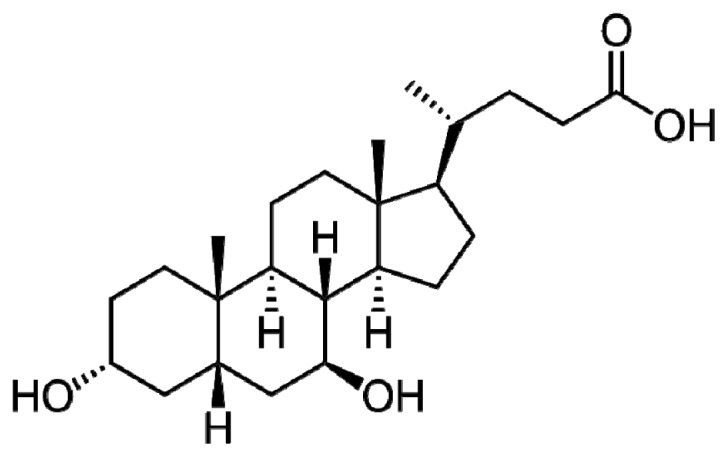
Structure of Ursodeoxycholic acid (UDCA).

UDCA is 3α,7β-dihydroxy-5β-cholan-24-oic acid (Figure 1), which is a secondary bile acid having hydrophilic properties. It is formed by 7b-epimerization of primary bile acid chenodeoxycholic acid in the gut by intestinal bacteria. Bile acids are steroid acids that emulsify intestinal lipids. Bile acids are derived from cytochrome P450- mediated oxidation of cholesterol. Bile acids are detergents, surfactants, interfere with protein-mediated hepatic long chain free fatty acid uptake and are potentially hepatotoxic [1,2,21,22]. UDCA is elevated during bear hibernation [23].

## 3. Potentially Toxic Molecular Properties of UDCA

### 3.1. UDCA Breaks Down into Toxic Lithocholic Acid

About 90% of a therapeutic dose of UDCA is absorbed in the small bowel after oral administration. After absorption, UDCA enters the portal vein and undergoes efficient extraction from portal blood by the healthy liver where it is conjugated with either glycine or taurine. UDCA in bile is concentrated in the gallbladder and expelled into the duodenum. Only small quantities of UDCA appear in the systemic circulation, in plasma UDCA is protein bound and very small amounts are excreted into urine [24].

Beyond conjugation, UDCA is not altered or catabolized appreciably by the liver or intestinal mucosa. UDCA is typically oxidized and reduced at the 7-carbon, yielding either 7-keto-lithocholic acid/or lithocholic acid, respectively. Lithocholic acid causes cholestatic liver injury and can cause death from liver failure in patients with compromised sulfation. Lithocholic acid induces DNA strand breakage, is uniquely co-mutagenic, promotes cell transformation, leads to segmental bile duct injury, liver cell failure and death [25,26]. In the normal subjects, 41% of ursodeoxycholic acid is 7-dehydroxylated to lithocholic acid during 2 h of incubation *in vitro* [27], and almost up to 100% *in vivo* in 12 to 24 h [28,29], with the major fecal bile acid of patients receiving UDCA is lithocholic acid.

UDCA half-life is appreciably long, estimated to be 3.5 to 5.8 days [30].

### 3.2. UDCA Inhibits Apoptosis, Arrests Cellular Regeneration, and Blocks DNA Repair

Apoptosis is the essential key process to remove damaged cells, maintain homeostasis of cell number, and is a process by which hepatic myofibroblasts disappear [31–33].

Once cells are damaged beyond capabilities of DNA repair pathways [34,35], they are sentenced to irreversible dormancy (senescence) [36–38], death (apoptosis), or undergo unregulated cell division (tumor formation). Once damaged and committed to apoptosis it exhibits thrombospondin binding sites, loss of sialic acid residues and phosphatidylserine on its cell surface signaling for neighboring phagocytic cells to commence phagocytosis and elimination of the apoptotic cell [39].

UDCA is anti-apoptotic [40,41]. UDCA anti-apoptosis is not limited to hepatocytes, as it is hydrophilic with greater systemic dissemination. UDCA anti-apoptosis is mediated via silencing of p53, inhibition of cyclin D1 [42], and through caspase independent mechanism [43]. UDCA also blocks apoptosis of damaged cells indirectly as well, through blocking deoxycholic acid cytotoxic bile acid induced apoptosis. UDCA suppresses DNA binding activity of activator protein-1 and leads to down-regulation of both extracellular signal-regulated kinase (ERK) and Raf-1 kinase activities stimulated by exposure to deoxycholic acid (DCA). DCA was also found to activate epidermal growth factor receptor (EGFR) activity and UDCA inhibited this. UDCA anti-apoptosis is partly mediated by molecular modulation of EGFR/Raf-1/ERK signaling [44]. Moreover, UDCA inhibits DCA-induced apoptosis in rat hepatocytes and nonhepatic cells *in vitro* by modulating mitochondrial membrane perturbation, reducing Bax protein abundance in mitochondria, as well as inhibiting reactive oxygen species [45].

**Figure 2:**
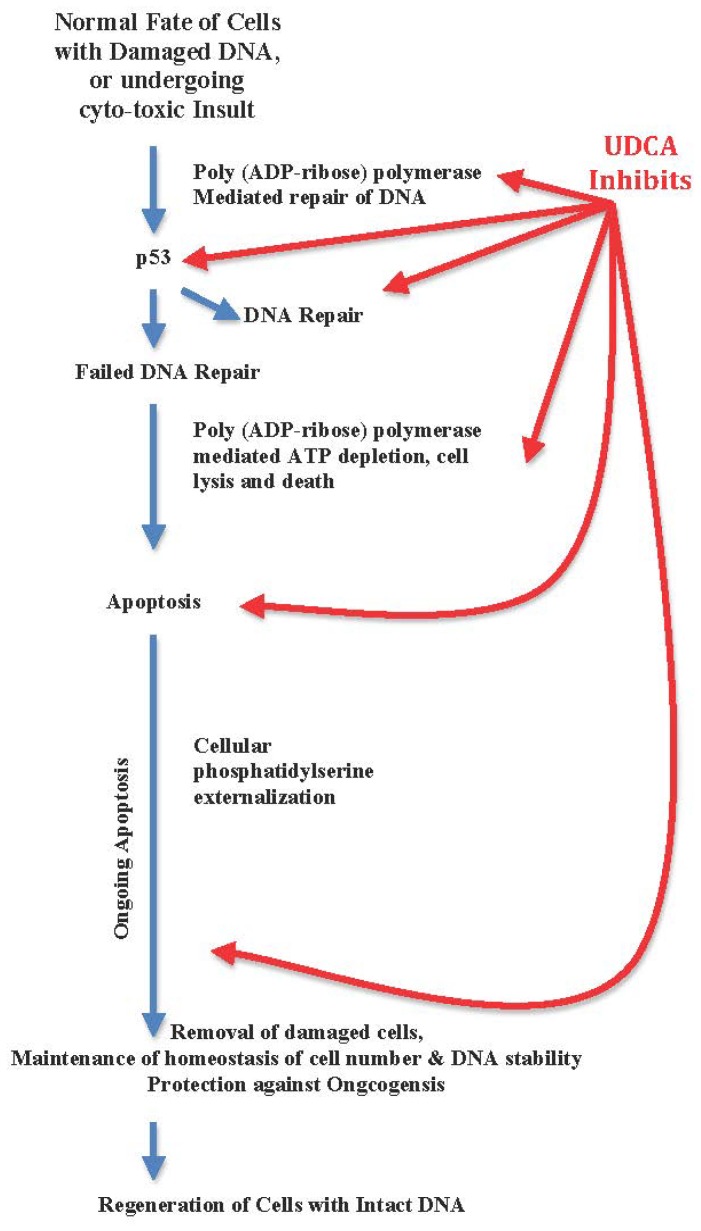
UDCA interferes with timely DNA repair and with apoptosis of DNA damaged cells with resultant mutagenic, and anti-proliferative properties.

UDCA modifies histone acetylation and induces differentiation and senescence [46,47]. The anti-apoptotic property of UDCA is effective even after phosphatidylserine externalization (Figure 2) [48]. In presence of UDCA, liver cells do not mount a cytoprotective cascade when confronted by cytotoxicity of bile acids [49,50]. UDCA interferes with the hepato-protective cytokeratin CK8 upregulation [51]. The anti-apoptotic property of UDCA is effective in hepatic and non-hepatic cells, and prevents apoptosis-associated alterations in mitochondrial transmembrane potential and reactive oxygen species production in the cultured cells to 0.5% ethanol acting through different apoptotic pathways [48].

The anti-apoptotic property of UDCA inhibits relentlessly a natural cascade of events that secure timely and effective regeneration of damaged cells.

Moreover, UDCA blocks poly (ADP-ribose) polymerase mediated DNA repair [19,52].

In Popov and Colleagues’ (2010) trial at inducing remodeling of liver fibrosis in rat liver, they concluded that the peak of connective tissue remodeling and fibrolytic activity was associated with massive apoptosis of cholangiocytes and their phagocytic clearance by macrophages *in vivo* [53].

It is crucial to emphasize that resistance to bile acid-induced apoptosis is tumorigeneic in human colorectal cancer. Inhibition of apoptosis results in increasing accumulation of DNA-damaged cells and results in increased cancer risk [40,54].

### 3.3. UDCA Inhibits Co-Enzyme A

Another façade for the orchestrated cellular handicapping of UDCA is made evident by inhibition of co-enzyme A. UDCA inhibits co-enzyme A dependent steps in the cholesterol degradation, and conjugation of bile acids [55]. Cholesterol is essential for building, maintaining, cellular membranes, and regulating their fluidity [56–58].

Cholesterol is the precursor of a cascade of steroid hormones, which define our daily bodily functions, structure and normal response to danger, of them sex hormones and adrenal gland hormones [59–61].

Not only does UDCA down regulate cholesterol synthesis, it arrests cellular response to cytotoxic stimuli being a most powerful inhibitor of adenosine 3′,5′-cyclic monophosphate (cAMP). cAMP is a second messenger of intracellular signal transduction, for hormones as glucagon and adrenaline, which cannot pass through the cell membrane. cAMP is involved in the activation of protein kinases, regulates the effects of adrenaline and glucagon, regulates the passage of calcium through ion channels, regulates glycogen, and other carbohydrate and lipid metabolism. UDCA specifically inhibit glucagon-induced cAMP synthesis mediated by protein C kinase [62–64].

### 3.4. UDCA Is a Transcriptional Factor

UDCA inhibits the dynamic and multiple functioned p53 [65,66]. The tumor suppressor protein, p53 is crucial for elimination of injured cells from the replicating pool to protect the organism from malignant transformation [65–67].

Engagement of the p53 signaling pathway occurs in response to a broad range of stressors, intrinsic and extrinsic to the cell, which stabilize and affect p53 by a series of post-translational modifications [68–72].

UDCA inhibits p53 induction and stabilization through a caspase independent mechanism. More importantly, bile acid inhibition of p53-induced apoptosis is associated with decreased p53 DNA binding activity. Subcellular localization of p53 is also altered by UDCA [65,66].

The expressions of p53 and pro-apoptotic Bax in hepatocytes are up-regulated by deoxycholic acid (DCA). This up-expression is inhibited by UDCA. The DCA-induced increased count of Bax-positive cells is reduced by UDCA. Transcriptional UDCA inhibition of DCA-induced hepatocyte apoptosis is by down-regulating the expression of p53/Bax signal molecule [43,65,73,74].

UDCA also inhibits degradation of nuclear factor kappaB (NF-kappaB) and its inhibitor kappaB [43].

Nuclear factor kappa (NF-kappa) is a protein complex that controls the transcription of DNA, and plays a key role in regulating immune response to infection, malignant transformation and processes of synaptic plasticity and memory [75–77]. UDCA suppresses NF-κB, through functional modulation of the glucocorticoid receptor and NF-kappa B dependent transcription [78].

While the UDCA nuclear factor kappa is seen as a potential anti-proliferative tool in malignant growths, UDCA promotes tumor genesis as it inhibits p53. Hence UDCA is believed to have a potential therapeutic application in cancer and inflammatory diseases [79,80]. This UDCA inhibition of nuclear factor kappa, needs more fine tuning in context of UDCA inhibition of induction and stabilization of p53, and also in the context that *up-regulation of anti-apoptosis* function of p73 (p53 homologue) subfamily is associated with reduced survival in hepatocellular carcinoma [81].

Bile acids except ursodeoxycholic acid up-regulate death receptor tumor necrosis factor-related apoptosis-inducing ligand (DR5/TRAIL)-receptor 2 expression via a c-Jun *N*-terminal kinase-dependent pathway [82].

Debate continues as the transcriptional abilities of UDCA, its interference with protein kinase C in various cell lines, and direct inhibition cholangiocyte proliferation is believed to promise a future role in control malignant disease [83–88].

Meanwhile, UDCA promotes tumor virulence, which is mediated by anti-apoptotic effect through silencing of p53, interference with protein C kinase, its anti-apoptosis and inhibition of NF-κB degradation [89,90]. UDCA role in malignant disease progression is novel and peculiar, and represents a class of its own as a mutational, and gene modifier class of possibly “tumor controlling” medication. The definitive clinical application of UDCA in prevention or treatment of malignancies remains to be established.

UDCA interacts with nuclear receptors that regulate gene-expression in a ligand-dependent manner, leading to conformational change that coordinately dissociates co-repressors and facilitates recruitment of coactivator proteins to enable transcriptional activation [91].

Moreover, UDCA inhibits histone acetyl transferases [92]. Histone acetyltransferases add acetyl groups to histones, allowing the tightly bound histone complex to relax and allow other proteins to act on the DNA. If histone acetyltransferases are inhibited, then damaged DNA may not be repaired, eventually leading to cell death [93].

UDCA other transcriptional effects are demonstrated by its immune-modulation and interference with detoxification.

### 3.5. UDCA Is Immune-Modulatory

UDCA is a steroid with immunomodulatory properties. UDCA suppresses production of IgM, IgG and IgA induced by Staphylococcus aureus Cowan I in peripheral blood mononuclear cells derived from healthy subjects and patients with primary biliary cirrhosis and also in human B lymphoma cell lines. UDCA also suppresses interleukin-2 and interleukin-4 production induced by concanavalin A and interferon-gamma production induced by polyinosinic-polycytidylic acid. UDCA suppresses the concanavalin A-induced thymocyte proliferation mediated by interleukin-1. It reduces the level of hepatic expression of human leucocyte antigens (HLA class I) [94,95], and does not have an effect on histamine release by mast cells [96].

Nuclear steroid receptors are ligand-activated transcription factors that play a key role in a variety of vital physiological phenomena including developmental or endocrine signaling, reproduction, and homeostasis. In addition, they are implicated in other important biological processes, such as apoptosis. UDCA as a cholesterol-derived molecule has chemical and structural similarities to steroid hormones, and being a bile acid, it modulates nuclear steroid receptor activation [97–100].

UDCA not only binds to glucocorticoid receptor, it leads to functional modulation of the glucocorticoid receptor as well, and suppresses the NF-kB-dependent transcription [43]. Sudden halt of UDCA results in a steroid withdrawal syndrome associated with rise of serum bilirubin and aminotransferases. The withdrawal syndrome is controlled by re-intake of UDCA [101].

Glucocorticoids interfere with prostaglandin synthesis and phospholipase A2 [102,103].

Again, this UDCA specific inhibition of prostaglandin A2, occurs at a transcriptional level [104]. UDCA activates the intracellular glucocorticoid receptor in a dose-dependent manner [99].

The UDCA enhanced glucocorticoid-induced tyrosine aminotransferase-gene expression is blocked by the transcriptional inhibitor (through inhibition of protein kinase C) sphingosine [105]. Glucocorticoid receptor and mineralocorticoid receptor, as well as the progesterone receptors and androgen receptors, bind in closely related ways to broadly overlapping response elements [106]. Steroids treatment allows glucocorticoid receptors to oligomerize with minerlocorticoids in the cytoplasm [107]. UDCA up regulates nuclear glucocorticoid and mineralocortcoids receptors [108], and down regulates progesterone and estrogen receptors [109]. UDCA modulates immune response by inhibition of mitochondrial membrane depolarization and channel formation, production of reactive oxygen species, release of cytochrome C, caspase activation, and cleavage of the nuclear enzyme poly adenosine diphosphate (ADP-ribose) polymerase [110]. UDCA also enhances natural killer cells activity in primary biliary cirrhosis patients [111]. UDCA in patients with cholesterol gallstones lead to a decreased number of activated macrophages in gallbladder muscle layer [112].

### 3.6. UDCA Interferes with Drug Metabolism and Detoxification

UDCA is a potent inducer of the cytochrome NADPH-CYP-c-reductase, Aminopyrine *N*-demethylase CYP3A1/2, p-Nitrophenol hydroxylase CYP2E1, Ethoxycoumarin *O*-deethylase, Pentoxyresorufin *O*-dealkylase CYP2B1/2, Methoxyresorufin O-demethylase CYP1A2, Ethoxyresorufin O-deethylase CYP1A1, and Lauric acid hydroxylase CYP4A and inhibits their inactivation [113], raises glutathione plasma levels albeit still subnormal or within normal levels [114], and induces multidrug resistance protein 3, but not multidrug resistance protein 4, and 5. UDCA ability to counteract bile acid toxicity is compromised [115].

Lipid soluble xenobiotics (drugs and compounds foreign to a human biochemistry) in need of detoxification freely diffuse across cellular membrane of hepatocytes. They undergo detoxification in a common pathway through phases I (detoxification), II (conjugation) and III (excretion). In phase I, hepato-cellular enzymes introduce reactive and polar groups to compound. This phase might result in activation of the xenobiotic being detoxified. Phase I involves a major contribution of the cytochrome P-450 super family oxidation. The introduced hydroxyl groups, or *N*-, *O*- and *S*-dealkylation of substrates result in electrophiles (acceptor of eclectrons, mostly positively charged) and nucleophiles (donors of electrons, *i.e.*, act as a base). Phase II scavenges the resultant of phase I, by active conjugation to glucouronic acid, sulfate, glutathione, and glycine [116–120]. Phase III is handled by the efficient membrane transporters family of the multi drug resistance protein. This family is involved with ATP-dependant transport of a huge variety of hydrophobic anions, to the extracellular matrix for further excretion or metabolism [121,122].

Xenobiotic timely coordinated detoxification is vital in infectious and malignant diseases development, management and prognosis [123].

UDCA accentuates phase I more than phase II, and does not influence all the multiple drug resistance proteins. Moreover, UDCA consumes glycine and conjugation of liver for its own detoxification and compete with other toxic xenobiotics, for phase II detoxification [25,26].

### 3.7. UDCA Is Choleretic

UDCA increases bile flow. However, the mere increase of bile flow in obstructive cholestasis without resolution of the cause may worsen the disease course due to increase of biliary pressure and leading to rupture of cholangioles and to development of bile infarcts. Obstructive cholestasis refers to obliterative and non-obliterative obstructions [124].

Non-obliterative small bile duct obstructions include late stages of PBC, primary sclerosing cholangitis, paucity of intrahepatic biliary radicals and vanishing bile duct syndrome [115,125].

Stimulation of bile flow even with the hydrophilic bile acid UDCA in a mouse model of sclerosing cholangitis and in bile duct ligated mice increased liver injury, aggravated bile infarcts and induced hepatocyte necrosis [124].

### 3.8. UDCA Is Hydrophilic

UDCA is a hydrophilic compound. Hydrophilic compounds escape diffusion within cell wall and escape detoxification, as most hydrophilic molecules cannot enter cells, since specific transporters do not recognize them [126].

They need specific detoxification systems as the glyoxalase system [117,127], and the other antioxidant systems that eliminate reactive oxygen species [128].

Hence, UDCA has a considerably long half -life. UDCA time to clearance from circulation is 3.5 to 5.8 days half-life [30], with a wide range of systemic effects and hepatic and extrahepatic toxicity.

### 3.9. UDCA Suppresses Central Nervous System Microglia Activation

UDCA effect on microglial cells arresting their potential, halting inflammation and compromising potential at repair, is another expression of UDCA generalized down-regulation of cellular functions. UDCA directly and effectively inhibits nitric oxide production by microglia cells. This effect on microglia is sustained up to 48 h [129–131]. UDCA microglial suppression and high penetrance of blood brain barrier promise a theoretical role in Alzheimer disease and amyotrophic lateral sclerosis [132,133].

Microglia are the resident macrophages of the brain and are the principal source of cytokines produced during central nervous system inflammation responsible for neuroprotection and immune surveillance [134,135].

They are responsible for cytotoxicity, antigen presentation, and synaptic stripping. They are pivotal for brain healing and repair. Microglial neuroinflammation result in neuroprotection, mobilization of neural precursors for repair, remyelination, and even axonal regeneration. Factors that adversely affect viability and self-renewal capacity of microglia, result in the generation of senescent and/or dysfunctional cells that contribute to the development of neurodegenerative disease and Alzheimer disease [136].

The outstanding example of consequences of microglia injury is unconjugated bilirubin toxicity to microglial cells that could be detrimental to the developing central nervous system in the neonate [137]. Failure of microglia entangled in overstimulation to move on to central nervous system regeneration is encountered in Alzheimer disease, cerebral malaria and Parkinson disease [138].

### 3.10. UDCA Anti-Proliferative Potential

UDCA is a unique molecule that arrests cellular and bodily functions, processes, metabolism and leads to “situational freeze”, or rather hibernation of the body. This situational freeze or hibernation is extremely evident by the clinical trials of UDCA in various diseases. UDCA never achieved cure in any disease entity, or in a double blind case controlled trial. Despite high hopes and tremendous expenditure, space and chance for UDCA to effect, it does not go beyond the slight initial improvement of surrogate markers [6–9].

UDCA did not affect human cervical carcinoma, breast cancer resistance protein or breast carcinoma cells, yet its synthetic derivative HS-1183 induces apoptosis in human cervical carcinoma cells [139–141].

UDCA induces a delay in cell cycle progression [142], and hepatocyte regeneration in response to cholera toxin [143].

UDCA significantly decreased the hepatocyte growth factor mRNA levels up-regulated by either phorbol-12-myristate-13-acetate or cholera toxin, partially inhibits up-regulation of hepatocyte growth factor gene expression (ranging from 40 to 50%), and inhibits cholera toxin-induced hepatocyte growth factor production to extent of more than 80% at 24 and 48 h [143].

Hepatocyte growth factor has been shown to play an important role in regeneration of various tissues and in embryonic and fetal development [144,145].

Hepatocyte growth factor has been shown to be effective in treating animal models of chronic hepatic and renal diseases such as hepatic and renal fibrosis and liver cirrhosis [146,147].

At high concentration, UDCA significantly inhibits cell proliferation, and is more anti-apoptotic, while it increases apoptosis at low concentrations. Tumor necrosis factor-alpha induced DNA fragmentation is potentiated by high dose of UDCA, but not by low and intermediate UDCA concentrations [148].

UDCA hypothetical room in malignant disease was supported by UDCA induced increase in the targeted apoptotic-associated cell death. By facilitating apoptosis over necrosis, UDCA increases the apoptotic and decreases the necrotic effects of SN-38 in the various adenocarcinoma cell lines, including HT-29. This effect of UDCA involves mitochondrial membrane depolarization and activation of caspase-3 and caspase-9 [149].

SN-38 is a potent metabolite that is thought to be a major player in the antitumor action of CPT-11 [150].

UDCA anti-proliferative effect awaits exploitation, when delivered systemically or locally as a single bolus as an adjuvant therapy for delivery of locally acting chemotherapy prior to debulking of a tumor, or prior to delivery of irradiation, or prior to needle aspiration or biopsy to arrest seedling.

### 3.11. UDCA Potentiates Cellular Cytotoxicity

UDCA is genotoxic as measured by micronuclei production, which is dose related and exerts aneugenic activity (leads to numerical chromosomal alteration). Micronuclei production in peripheral blood lymphocytes is a biomarker of chromosomal damage for genotoxicity testing and biomonitoring studies [151].

Micronuclei derive from chromosomal fragments and whole chromosomes lagging behind in anaphase [152].

Tumor necrosis factor-alpha induced DNA fragmentation is potentiated by high dose of UDCA, but not by low and intermediate UDCA concentrations [148].

UDCA leads to remarkable hepatic atrophy associated with many focal areas of necrosis and hepatotoxity in mice treated with griseofulvin, that was not evident in the mice treated with griseofluvin alone [153].

UDCA potentiates photodamage in leukemia cells. Photodamage causes a rapid loss of the mitochondrial membrane potential, loss of cytochrome *c*, and initiation of a prompt apoptotic response. UDCA potentiated loss of mitochondrial potential, release of cytochrome *c* into the cytosol, activation of caspase-3, and apoptotic cell death after irradiation of photosensitized murine leukemia L1210 or hepatoma 1c1c7 cells. These effects were not observed when UDCA was added after irradiation [154].

UDCA potentiates chenodeoxycholic acid cytotoxicity, probably at the level of induction of the mitochondrial permeability transition, and decrease in mitochondrial membrane potential and depletion of ATP. In the presence of UDCA, chenodeoxycholic acid-induced apoptosis is not properly executed but degenerates into necrosis [155].

### 3.12. UDCA Controls Bilirubin and Hepatic Transaminases

UDCA achieves a 25% drop in serum bilirubin, a 35% drop in serum alanine aminotransferase, a 33% drop in aspartate aminotrasferase, 40% drop in alkaline phosphatase and a 50% drop in gamma glutamyl transpeptidase, that is not associated with control of pruritus or weakness. UDCA was reported to lower serum bilirubin and hepatic transaminases [13,156]. In patients with PBC on UDCA, the mean serum bilirubin was 1.58 mg% (range 1 mg%–2.1 mg%), compared to the control group total bilirubin mean 2.26 mg% (range1.6 mg%–4.6 mg%) [6–9].

UDCA produces hypercholeresis that appears attributable to stimulation of biliary bicarbonate output and is decreased or abolished in the perfused rat liver by amiloride or perfusate Na^+^ substitution. These and other findings indicate that UDCA hypercholeresis is tightly linked to biliary excretion and suggest that UDCA biotransformation may be influenced by intracellular pH [157].

Moreover, corticosteroids exhibit decrease in serum bilirubin when used in patients with hyperbilirubinemia. UDCA and corticosteroids lowering effect on bilirubin is independent of objective improvement in pathology [13,158–160].

Moreover, decrease of liver transaminases is not synonymous to improvement in chronic liver diseases. Patients with cirrhosis and poorest prognosis among patients with chronic liver disease have the lowest hepatic transaminase levels [161]. Higher ALT was reported as better prognostic marker of hepatitis B virus clearance [162], and a normal ALT as a surrogate marker in chronic hepatitis B virus infection is of questionable value [163]. Control of ALT was achieved in a cohort of 41 patients with concomitant HIV and HCV during treatment with interferon, yet only one single patient achieved sustained HCV clearance [164], while a higher ALT was reported in a study of natural history of HIV and HCV where 15% of patients cleared the HCV load spontaneously [165]. Hepatic transminases are overrated as prognostic or diagnostic tools. Acute hepatitis with best prognosis [166,167] has high transaminases [168], and low ALT is associated with development of cirrhosis in autoimmune hepatitis [169]. While evidence objectively refutes correlation of control of ALT with better prognosis [170,171] a higher ALT is prognostic of better outcome and HBV and HCV clearance and response to therapy [162,172]. In fact, control of transaminases was the outstanding feature of patients with primary sclerosing cholngitis receiving UDCA in the trial recently halted in North America. This cohort suffered from detrimental liver cell failure and death [13]. In fact, the “controlled” transaminases should be viewed in context of UDCA down regulation of cellular functions, inhibition of DNA repair [42,43], inhibition of hepatocyte growth factor [143] and inhibition of cellular proliferation as an alarming sign.

## 4. UDCA as a Medicine

Beyond primary biliary cirrhosis, lack of evidence-based effectiveness of UDCA in liver disease testifies of how misplaced UDCA is as a medicine. UDCA does not cure cholestasis, or liver disease [8,156,173,174]. UDCA properties promise a class of its own as a transient anti-metabolite, and a tumor suppressive agent. Long term follow up of patients enrolled in short term studies of UDCA is mandatory to study long term effects of its transcriptional effects, p53 inhibition, and production of lithocholic acid as clearly depicted by case-control trial recently halted in North America, post-marketing statistics and the retrospective studies [8,9,11–13].

### 4.1. UDCA in Primary Biliary Cirrhosis

Primary biliary cirrhosis is an immune mediated disease. UDCA was employed as a choleretic and immune-modulatory tool. Reports have demonstrated a unanimous decline of aminotransferases, alkaline phosphatase, and serum bilirubin, independent of histological improvement. Debate remains as regards UDCA effectiveness in terms of survival, need for liver transplantation, or histological improvement [8]. UDCA is approved for treatment of PBC [3,5]. The much-generated debate of whether UDCA affects transplantation free survival and death is highlighted in review articles and meta-analyses, where all agreed upon some control of biochemical parameters in UDCA receiving patients, while the effect on histopathology and transplantation free survival was not reproducible in studies, and if detected was limited to those with asymptomatic PBC. However, the fact lies that UDCA is not a curative treatment of PBC, and it does not halt disease progression [133,175–180].

Moreover, asymptomatic cases have a favorable natural history, and cannot be considered within same cohort with symptomatic PBC. It is to be noted specifically that two out of three asymptomatic primary biliary cirrhosis patients benefit from UDCA use [181,182] compared to equal or better natural history with reports of 57%, 70%, and more than 90% 10 years patients survival without medications [183–185]. Again, the expected median survival of asymptomatic PBC patients was 10 and 16 years in two large cohorts followed for up to 24 years [186,187], while median survival of symptomatic patients is approximately seven years [187,188].

The systematic review of 16 randomized clinical trials evaluating UDCA in primary biliary cirrhosis against placebo or no intervention demonstrated no significant effects favoring UDCA on mortality and mortality or liver transplantation. UDCA effects do not go beyond some improvement of serum levels bilirubin, hepatic alanine and aspartate aminotransferase, alkaline phosphatase. It was found that UDCA did not improve pruritus, fatigue, autoimmune conditions, liver histology, or portal pressure. The use of UDCA was significantly associated with adverse events, mainly weight gain [8]. The reported UDCA 25% drop of bilirubin level is of statistical significance, but is not of clinical significance, and does not go beyond improvement of surrogate markers [8,9,175,183–185,189]. It is to be noted however that steroids exert same effect on liver biochemical markers in PBC [190]. Moreover, the risk of development of hepatocellular carcinoma in patients with PBC who do not respond to UDCA biochemical control increases by time, with a 10 and 15 years incidence of 9% and 20% respectively [16].

### 4.2. UDCA in Primary Sclerosing Cholangitis

UDCA failed in controlling liver histology, or symptoms, in a dose of 13–15 mg/kg/day [191,192]. In a higher dose of 28–30 mg/kg/day, UDCA resulted in more than double fold increase in patient deaths, and need for liver transplantation. There is an exceptionally narrow difference between recommended dose and detrimental dose of UDCA in primary sclerosing cholangitis [13,26].

### 4.3. UDCA in Pediatric Cholestasis

In pediatric age groups there is no evidence from case controlled double blind studies that indicate any curative role of UDCA in neonatal hepatitis, paucity of intrahepatic biliary radicals, extrahepatic biliary atresia and primary sclerosing cholangitis [11,12,189,193]. Moreover, serious life threatening complications, and failure of cholestasis resolution were more than double fold in those receiving UDCA, necessitating halt of off-label use and clinical trial in infants and children in Cairo University Children Hospitals by Higher Committee For Medications-Cairo University Hospitals in 2010. Liver sulfation and conjugation physiology in children is not congruent with that of adults [194–197]. UDCA use in children is entirely not evidence based. In view of its carcinogenicity, it is advisable that UDCA be strictly used in pediatric age groups within sound clinical trials.

### 4.4. UDCA in Hepatitis B, and C Virus Infection

There is insufficient evidence either to support or to refute the effects of bile acids on long-term outcomes including hepatocellular carcinoma, hepatic decompensation, and liver related mortality in patients with hepatitis C or B viral infections. Randomized trials with high methodological quality are required before clinical use is to be considered [198].

### 4.5. UDCA in Cystic Fibrosis

Whereas the life expectancy of patients with cystic fibrosis (CF) has been increasing, associated liver disease has emerged as a significant medical issue affecting almost 7–9.7% of adult patients with cystic fibrosis [199,200].

The liver affection in patients with cystic fibrosis ranges from mild hepatitis to cirrhosis and portal hypertension [201].

UDCA may improve biochemical parameters of liver disease, but not steatorrhea in cystic fibrosis patients. Evidence is lacking as regards the effect of UDCA on histopathology of liver disease in cystic fibrosis [202,203].

UDCA long-term efficacy in preventing the progression of liver disease in CF is unproven [204,205].

Moreover, UDCA is not effective in cholesterol gall stone dissolution in cystic fibrosis patients [206]. Meta-analyses of UDCA in cystic fibrosis concluded that there was insufficient evidence to justify its routine use in cystic fibrosis [174,207].

### 4.6. UDCA in Non-Alcoholic Steato-Hepatitis

Meta-analyses of four randomized clinical trials randomizing 279 patients could not find significant differences regarding mortality or improvement in liver function tests observed after treatment with UDCA. Data on the radiological and histological responses were too scant to draw any definite conclusions [208]. High dose UDCA failed to improve the overall histology in 185 patients with NASH in comparison with placebo [209].

### 4.7. UDCA in Gall Stone Dissolution

The standard treatment of symptomatic gallstone subjects is laparoscopic cholecystectomy. Only patients amenable for nonsurgical therapy with mild symptoms and small, uncalcified cholesterol gallstones in a functioning gallbladder with a patent cystic duct are considered for oral litholysis by hydrophilic ursodeoxycholic acid, in the hope of achieving cholesterol desaturation of bile and progressive stone dissolution [210]. As not all cholesterol gallstones are symptomatic, UDCA is of questionable value.

### 4.8. UDCA in Cancer Colon

Phase III Trial of UDCA in colorectal neoplasia, during a 6 to 36 months, follow up failed in reduction of incidence but reduced grade of the dysplasia of recurring lesions from 8.7% among the placebo group to 5.5% among the UDCA group. Long-term outcome of UDCA remains to be identified [165,211,212].

## 5. UDCA Associated Adverse Drug Reactions

UDCA associated adverse drug reactions are related to its properties. It leads to immune suppression and consequent fever, pneumonia, pharyngitis, otitis media, bronchopneumonia, bronchitis, oral moniliasis, abscess formations, dysuria and recurrent watery diarrhea. Long standing use of UDCA is recognized to be associated with tubulointerstitial nephritis, leukocytoclastic vasculitis, skin rash, thrombocytopenia, recurrent wheezy chest, cough and interstitial lung disease. Its use is also associated with hepatic complications as vanishing bile duct syndrome, pruritus, cholangitis, ascites, increasing cholestasis, portal hypertension and liver cell failure. Other reported miscellaneous adverse drug reactions included convulsions, nausea, vomiting, sleep disturbance, diabetes and cancer breast [12,13,20,213].

## 6. Why Were Ineffectiveness and Deaths Associated with Double Dose UDCA Thought to Be “Unanticipated”?

UDCA, is of unproven effectiveness in cholestasis, acute or chronic liver disease, colorectal carcinoma, and has specific molecular toxicity. It freezes regeneration and induces cellular hibernation. No case control double blind trial has demonstrated its true curative effects in any liver disease.

Despite high hopes and tremendous expenditure, space and chance for UDCA to effect, it does not go beyond “cellular freezing”, and arrest of cellular regeneration.

The major cofounder however remains to be the insinuation of a “hepatoprtective” effect of UCDA.

### 6.1. “Hepatoprotective” as a Term Is Not Tangible, Not Objective, Is Immeasurable, Not Quantifiable, and with Unknown Sequel or Clinical Effects, or Benefits, and Is of No Prognostic Value

“Hepatoprotective” needs definition before being applied as an indication for drug intake. It encouraged wide UDCA off-label use and disseminated the unforeseen mutagenic and hepatotoxic UDCA. UDCA unique anti-proliferative properties promise a future role for UDCA as an adjuvant bolus therapy in malignant disease management and hibernation science.

### 6.2. Relying on False Surrogate Markers

Serum bilirubin and transaminases cannot predict UDCA hepato-toxicity. They are not surrogate markers of liver histopathology.

### 6.3. Overrating UDCA Hydrophilic Properties & Underestimating Its Tight Therapeutic Index

Being hydrophilic allows a longer half-life and systemic dissemination [30]. The very narrow therapeutic index of UDCA (difference between recommended dose and lethal dose) warrants limiting its use as a medicine to approved indications only and as an investigational tool to clinical trials with strictly accurate objective endpoints and not surrogate biochemical markers.

## 7. Lessons to Be Learned. What Masked UDCA Toxicity?

### 7.1. Clinical Trial Flaws and Bias

1. UDCA is biologically transformed into lithocholic acid that induces DNA strand breakage. It is uniquely co-mutagenic, promotes cell transformation, and leads to liver cell failure and death. Clinical Trials of UDCA did not uniformly include measurements of lithocholic acid [13,26].
2. Primary biliary cirrhosis is a rare disease with a long course, spanning a lifetime. Some clinical trials of UDCA lacked a control group, or resorted to computer-generated controls [183–185,214].
3. UDCA results in a drop of 25% of serum bilirubin in patients compared to control groups. Though a 25% drop is of statistical significance, it is of marginal clinical and prognostic significance [8].
4. UDCA clinical trials depended on hepatic transaminases and serum bilirubin control as surrogate markers of effectiveness of UDCA. Liver transaminases and serum bilirubin are not surrogate markers of liver histopathology, and do not rule out underlying fibrosis, cirrhosis, or malignancy [215].
5. UDCA is an RNA transcriptional factor. Not all clinical trials of UDCA were long enough beyond 2 years to foresee the full-blown effects of UDCA [8].
6. Inclusion of asymptomatic cases of primary biliary cirrhosis and symptomatic cases as single cohorts is a major confounder. Asymptomatic primary biliary cirrhosis patients have a reported 57–90% 10 year survival [183–185,216].
7. Drawing conclusions from comparison of heterogeneous cohorts of asymptomatic cases, which have an incidence of future hepatic involvement of only 10% to an already affected population with cystic fibrosis, as a measure of pre-emptive treatment effectiveness [199,207,217,218].

### 7.2. Undermining Evidence

1. Highlighting UDCA’s down-regulation of cellular functions of hepatocytes, phagocytes and micoglia, and anti-apoptosis as an *advantage* while apoptosis eliminates DNA damaged cells beyond repair. Anti-apoptosis is a virulence factor of hepatitis C virus, and the virulence factor of hepatocelluar carcinoma [31–33,219–221].
2. Highlighting UDCA’s novel immune modulatory role, while UDCA is a steroid, which reacts with glucocorticoid receptors and retains immune-suppressive effects [94,95,97–100].
3. Describing the use of UDCA as “widely used”, while UDCA approved use is strictly limited to primary biliary cirrhosis, and gall stone dissolution. UDCA is not licensed for use in children [10].
4. Insinuation of a “hepato-protective” role of UDCA as an indication for its use, when evidence-based medicine proved that UDCA has no effect on histopathology or morbidity or mortality or quality of life. Moreover, in trying to force an increasing dose to allow for more “hepato-protection”, patients suffered more than double fold deaths [13,26].
5. Misinterpreting UDCA sudden withdrawal syndrome, and its dependency in favor of UDCA [101,222].
6. Propagation of the hydrophilic property of UDCA as an advantage. It is important to note that the more hydrophilic the compound is, the more it escapes detoxification and allows systemic dissemination and longer half-life [223–225]. Thus, UDCA half-life is 3.5–8 days, and it leads to suppression of central nervous system microglia activation [30]. It is important to highlight that micoglia injury by bilirubin intoxication in neonates is detrimental to developing central nervous system in neonates [137].
7. Lithocholic acid assessment is generally disregarded in trials of UDCA despite the well-known typical oxidation and reduction at the 7-carbon, yielding either 7-keto-lithocholic acid/or lithocholic acid, respectively [25,226].

### 7.3. Confounders

1. Under representation of review articles written by authors with no conflict of interest, and an abundance of review articles written by authors acknowledging conflicts of interest in leading journals of high impact factors [227].
2. Cofounding investigational use of UDCA with UDCA approved use as a medicine encourages off-label use in un-approved indications [22].
3. Use of the term “hepato-protective” that lacks objective definition. This insinuation encourages widening of scope of off-label use of UDCA to unapproved indications. This insinuated term shaped a “cultural” room for UDCA, when it failed to earn evidence based room as a curing medication [8]. The word ‘Hepatoprotective’ needs definition before being applied as an indication for drug intake. It encouraged wide UDCA off-label use, and disseminated the unforeseen hepatotoxic UDCA.
4. Over magnification of theoretical role of UDCA [227,228], and of its cultural root as a remedy ignoring the fact that there is limited evidence based effectiveness, propagated use of UDCA despite the lack of objective evidence on effect on mortality, histopathology or patient survival.
5. UDCA’s very narrow therapeutic index (difference between safe and toxic dose) warrants limiting its use to clinical trials with strictly accurate objective endpoints and not surrogate biochemical markers [26,226].
6. Prescribing habits are not congruent with scientific evidence. Approximately 91% of gastroenterologists in UK prescribe UDCA to primary biliary cirrhosis patients despite conflicting evidence of UDCA effectiveness or safety [229].

## 8. Conclusions

UDCA is a unique molecule that is cytotoxic, anti-proliferative, inhibits cellular regeneration, suppresses the immune system, and inhibits p53. UDCA hepatotoxicity in neonatal cholestasis in Cairo University Children’s Hospital, Egypt and in double dose in primary sclerosing cholangitis might be the tip of the ice-berg hitting our conscious, and challenges our understanding of the molecule. Off-label use of UDCA should be discouraged. Timely publication of phase IV post-marketing trials of UDCA is of prime importance.

UDCA use as a medicine should be strictly limited to approved indications and its investigational use should be limited to double blind case control clinical trials, that include assessment of lithocholic acid, and depend on real histopathology, morbidity and mortality outcomes and not on computer generated controls or false surrogate markers.

Conclusions drawn from studies depending on false surrogate markers and computer-generated controls, should be guarded and limited.

Exploitation of UDCA molecular toxicity and molecular properties as a hibernation agent, as a sclerotic agent and adjuvant cytotoxic agent for local arrest of proliferation of tumor beds after debulking or at biopsy sites await future verification.
